# Premature ejaculation - current concepts in the management: A narrative review

**DOI:** 10.18502/ijrm.v19i1.8176

**Published:** 2021-01-25

**Authors:** Arkiath Veettil Raveendran, Ankur Agarwal

**Affiliations:** ^1^Govt Medical College, Kozhikode, Kottayam, Manjeri, Kerala, India.; ^2^Seceratory General for Asia Oceania Federation of Sexology, Hon. Treasurer for CSEPI, Sex Counselors and Therapists, Indore, India.

**Keywords:** Premature ejaculation, Cognitive behavioral therapy, Serotonin uptake inhibitors, Dapoxetine.

## Abstract

Premature ejaculation (PE; early ejaculation or rapid ejaculation) is a common sexual problem affecting about 20-30% of men in the sexually active age group. PE can be of four types: Primary, secondary, natural variable, and subjective PE. Various non-pharmacological and pharmacological treatment options are available to treat PE including Dapoxetine, which is specifically developed for the treatment of PE. In this review, we discuss the pathophysiology and management aspects of PE.

## 1. Introduction 

Premature ejaculation (PE) is a common sexual problem encountered by men in day-to-day clinical practice. It affects about 20-30% of men in the sexually active age group leading to psychological stress and loss of self-esteem, resulting in significant adverse effects on the quality of life, of both the patient and the partner. PE is a commonly used term, but it is more appropriately called early ejaculation or rapid ejaculation.

### 1.1. Definition 

Although PE was reported in the medical literature years back in 1887, its first acceptable clinical definition was proposed in 1970 by Masters and Johnson as “the inability of a man to delay ejaculation long enough for his partner to reach organism on 50% of intercourse attempts” (1).

PE is defined by The American Urology Association as “ejaculation occurring sooner than desired causing distress to one or both partners” (2).

The International Society for Sexual Medicine (ISSM) defines PE as:

“A male sexual dysfunction characterized by ejaculation which always or nearly always occurs prior to or within about one minute of vaginal penetration (lifelong PE), or, a clinically significant and bothersome reduction in latency time, often to about 3 minutes or less (acquired PE), and; inability to delay ejaculation on all or nearly all vaginal penetrations; and negative personal consequences, such as distress, bother, frustration, and/or the avoidance of sexual intimacy” (3).

## 2. Physiology of ejaculation

**Figure 1:**
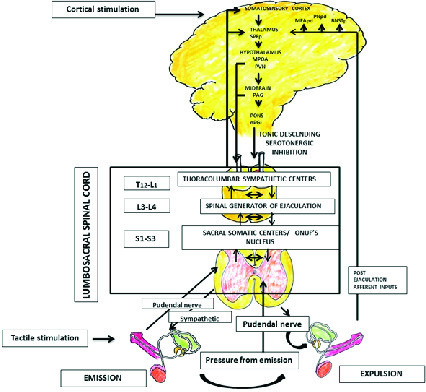
Physiology of ejaculation (see text for explanation). BNSTp: Posteromedial bed nucleus of stria terminalis; MEApd: Posterodorsal medial amygdaloid nucleus; MPOA: Medial preoptic area; PAG: Periaqueductal grey; nPGi: Paragigantocellular nucleus; PNpd: Posterodorsal preoptic nucleus; PVN: Paraventricular thalamic nucleus; SPFp: Parvicellular part of the subparafascicular thalamus (4, 6, 8, 9).

Ejaculation is the process by which sperms moves from epididymis via vas deferens to urethra and finally expelled out through the urethral meatus by the contraction of smooth muscles. A normal antegrade ejaculation consists of emission, expulsion (ejection), and orgasm (4, 5) (Figure 1).

Emission is the process of ejection of spermatozoa along with the products of accessory sexual gland secretion into the posterior urethra by smooth muscle contraction. The organ involved in emission phase includes epididymis, vas deferens, seminal vesicles, prostate gland, prostatic urethra, and bladder neck. These structures are supplied by both sympathetic and parasympathetic nerves derived from pelvic plexus. The closure of bladder neck during emission phase prevents retrograde ejaculation. Emission and closure of bladder neck are mediated by thoracolumbar sympathetic events which are mainly alpha adrenergic.

Expulsion is the process of ejection of sperms from the urethra through the urethral meatus/glans meatus. It is a spinal cord reflex occurring at the “point of no return” by the contraction of the smooth muscle of bladder neck, pelvic floor, bulbospongiosus, and ischiocavernosus in a stereotypical rhythmic pattern. The organ involved in expulsion includes bladder neck and urethra, and pelvic floor muscles. The smooth muscles of bladder neck and proximal urethra receive both sympathetic and parasympathetic supply. The external urethral sphincter and pelvic floor muscles receive somatic nerve supply. The muscles involved in expulsion phase are innervated by pudendal nerve and the motor neurons located in the nucleus of Onuf (ON). Normal pelvic floor function is important for normal sexual function. The superficial layer of male pelvic floor consists of bulbospongiosus, ischiocavernous, superficial transverse perineal muscle and external anal sphincter. These muscles are important in the control of urination, ejaculation, penile rigidity, and hardness during erection (6).

There are two central pattern generator for ejaculation, one for emission phase which is located in the upper lumbar cord (L2/L4) and the other for expulsion phase located in upper sacral cord (probably in S1/S2). Ejaculation is a reflex activity which is influenced by cerebral control. Spinal ejaculation generator act as a modulator of the emission and expulsion phase of ejaculation by coordinating genital sensory inputs, motor outputs, and descending supra spinal modulation from brain region (7).

A “spinal ejaculatory generator” integrates peripheral and central stimuli and its function is important in the process of ejaculation. The spinal ejaculation generator is under the influence of supraspinal area including nuclease paragigantocellularis, paraventricular nucleus of hypothalamus, and the medial preoptic area. The projection from the paraventricular hypothalamic nucleus terminates in the lumbar preganglionic motor neurons, nucleus of ON, sacral parasympathetic motor neurons, and neurons in the area of lamina X, where ejaculation generators are supposed to be located. Serotonergic projections from the nucleus paragigantocellularis (nPGi) in the brain stem exert tonic inhibition of ejaculation via motor nucleus in the lumbosacral spinal cord, influencing the spinal ejaculation generator in the spinal control center located in the lumbosacral spinal cord.

Ejaculation involves various cerebral and spinal areas which are interconnected. The various neurotransmitters involved in ejaculation include serotonin (5HT), dopamine, oxytocin, gama-aminobutyric acid (GABA), adrenaline, acetylcholine, and NO. Among these, 5HT has a primary role in ejaculation and is mainly inhibitory. 5HT${}_{1A}$, 5HT${}_{1B}$, 5HT${}_{2A}$, and 5HT${}_{7}$ receptor subtypes are mainly involved in the control of ejaculation. The neurotransmitter serotonin (5 Hydroxytryptamine-5HT) in the brain descending pathway inhibits ejaculation reflex. Activation of postsynaptic 5HT receptors, by 5HT in the synaptic cleft increases ejaculatory latency.

**Figure 2:**
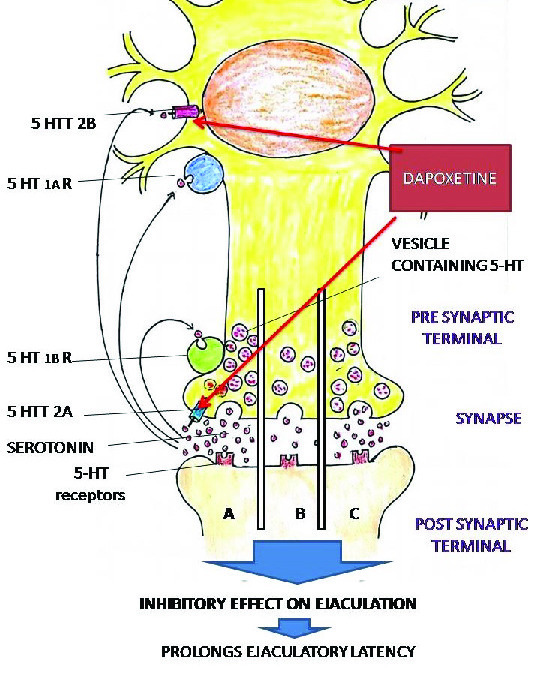
Mechanism of action of Dapoxetine in PE. (A) Synapse showing serotonin in normal individual. (B) Synapse showing reduced serotonin in people with PE. (C) Synapse showing improved serotonin level in people with PE treated with dapoxetine. 5HT: 5 Hydroxytryptamine or Serotonin.

Overstimulation of postsynaptic 5HT receptor is checked by three inhibitory mechanisms: (1) Activation of presynaptic 5HT${}_{1A}$ receptors, which reduces the firing rate of the neuron leading to inhibition in the release of 5HT into the synaptic cleft. (2) Activation of 5HT${}_{1B}$ receptors that reduce the release of 5HT in the synaptic cleft. (3) 5HT reuptake transports (5HTT), located at the presynaptic terminals and serotonergic cell bodies remove 5HT from the synaptic cleft into the presynaptic neurons (8) (Figure 2).

Studies have shown that during ejaculation, the strongest activation occurs in the meso-diencephalic region. The strongest activation seen in structures like ventral tegmental area (reward area), ventromedial posterior thalamic nucleus, subparafasicular nucleus, intralaminar nuclei, and lateral central tegmental field). Other areas showing increased activation include lateral putamen, parts of prefrontal, temporal, parietal, and insular cortex and cerebellum. The medial part of the amygdale region, which constantly monitors the environmental stimuli is deactivated during sexual activity (9).

## 3. Pathophysiology

Serotonin (5 Hydroxytryptamine-5HT) inhibits ejaculation reflex leading to prolonged ejaculation latency time. 5HT receptor agonists (5HT${}_{2c}$) also delay ejaculation (10). However, the low level of 5HT neurotransmission/hyposensitivity of postsynaptic 5HT receptors in brain stem/spinal cord results in low inhibition, resulting in rapid ejaculation. Genetic predisposition resulting in impairment of serotonergic pathway can also cause PE (Figure 2).

According to the Waldinger's theory of pathophysiology of PE, low levels of 5HT/hyposensitivity of 5HT2c receptor or hyper sensitivity of 5HT1A receptor cause PE.

In males, the pelvic floor dysfunction is associated with various sexual problems including erectile dysfunction ED and sexual dysfunction associated with ejaculation and orgasm.

Active perineal muscle control can inhibit ejaculation reflex via relaxation of bulbospongiosus and ischiocavernous muscles (11).

To summarieze, 5HT is the major neurotransmitter involved in the process of ejaculation and inhibits ejaculation reflex. Thus, a low level of 5HT or 5HT receptor hyposensitivity leads to PE, and drugs that increase the level of 5HT are useful in the treatment of PE.

## 4. Causes of PE

While PE is a common sexual dysfunction affecting all age groups, ED is more prominent in older age group and more commonly associated with other medical conditions like diabetes.

Primary PE can be triggered by psychological impulses like conditioning, upbringing, or traumatic sexual experience. Secondary PE can be triggered by diabetes, hypertension, hyperthyroidism, alcoholism, or use of recreational drugs. Psychological issues like depression, stress, or anxiety about sexual performance can also trigger PE. In addition, hyperthyroidism is associated with PE and treating the same results in improvement of PE. PE is common in patients with substance abuse. Besides, lack of sleep can lead to low serotonin, also resulting in PE.

## 5. Clinical features

PE is a self-reported problem, which not only affects the patient, but also the partner, and it also depends on the patient's expectation and belief regarding sexual intercourse. Hence, people who are able to perform sexual activities for reasonably good duration of time may also complai of PE due to unrealistic expectation about sexual activity. Measurement of intravaginal ejaculation latency time (IELT) is an attempt to define PE in terms of time taken to ejaculate, but it will not take into consideration the consequent psychological difficulty and distress, which are the main factors determining the quality of life and compelling the patient to seek medical attention. IELT is defined as the time interval between vaginal penetration and intravaginal ejaculation which is usually measured using a stopwatch and showed a positively skewed distribution with a median IELT of 5.4 min (0.55-44.1m) which decreases with age (12).

Sexual assessment monitor (SAM) is a novel portable device which measures time to erection and ejaculation. The device has a control box, a vibrator, and a sensor. The vibrator and sensor are attached to the penis. The vibrator helps to provides a non-manual stimulation whereas the sensor measures the time to erection and ejaculation. SAM generates ELT from electronically collected data. This helps in the objective measurements of ELT, resulting in more appropriate diagnosis of PE (13).

In patients with PE, we have to consider the time taken to ejaculate (ejaculate latency), the ability to control ejaculation, and the psychological (frustration) distress and its impact on the quality of life. The partner's attitude and the intersexual relationship between the couple should also be assessed. Patients with PE may have a tendency to avoid sexual intercourse, in order to escape from the psychological distress.

The inability to delay or control orgasm is considered to be an important factor as majority of patients with PE reports poor/ very poor ejaculatory control. But However, a number of studies have demonstrated only moderate or no correlation between IELT and reported ejaculatory control (14). PE causes psychological distress, anxiety, embarrassment and depression, erectile dysfunction, reduced libido, poor interpersonal relationship and, difficulty in relaxation and anxiety about intercourse. It also causes low scoring in self-esteem and relationship questionnaire, in all measures of intimacy on psychological and interpersonal relationship scale, greater distress on PE profile scale, decreased self-esteem and confidence during sexual encounters (15, 16).

Although the currently proposed definition focusses only on vaginal penetration, it fails to address other sexual activities like oral/anal sex and masturbation. However, in these sexual activities, sometimes partner's orgasm may not be an important factor, thereby reducing the clinical significance and associated psychological stress.

### 5.1. Classification of PE

PE can be classified into four major types: (1) Primary/life-long PE; (2) secondary/acquired PE; (3) natural variable PE; and (4) subjective PE/premature-like ejaculatory dysfunction. A primary PE is a form of PE which begins when a male becomes sexually active, while a secondary PE begins later in sexual life.

While a primary or Life-long PE is characterized by an IELT of <1 min since first intercourse, an acquired or secondary PE usually has an IELT of < 3 min at any given time (17). On the other hand, a variable PE is a normal variant of sexual function. In premature-like ejaculatory dysfunction, a person is preoccupied with early ejaculation or lack of ejaculatory control, but the IELT will be within normal limit (18) (Table I).

Moreover, PE can be divided into global or situational, depending the circumstances, and further divisions can be made depending on the type of sexual activity. Another classification of PE depending upon the etiology is “psychogenic PE” and “biogenic PE”.

Erectile dysfunction can coexists with PE, as man with ED ejaculate early, before the loss of erection; and identification of this situation is important in the management (19, 20). The loss of erection before ejaculation helps to differentiate ED from PE.

**Table 1:** Types of premature ejaculation and its features


**Features** **Types of PE**	**Lifelong PE**	**Acquired PE**	**Natural variable PE**	**Premature-like ejaculatory dysfunction/ Subjective PE**
**Possible etiology**	Genetic and neurobiological factors	Psychological and relationship problems, Sexual performance anxiety, Withdrawal/detoxification from prescribed or recreational drugs, Medical problems like • Urological (ED, prostatitis) • Endocrine (hyperthyroidism)	Normal variance of sexual performance	Cultural or psychological preoccupation with imagined rapid ejaculation
**Prevalence in general population (%)**	2.3-3.2	3.9-4.8	8.5-11.4	5.1-6.4
IELT*	Within 30-60 sec or between 1 and 2 min	Short (less than 2 min)	Short or normal	Normal range or may even be of longer duration
**Onset**	From about the first sexual encounter	At some point in a man's sexual life	Inconsistent and occur irregularly	Subjective perception of consistent or inconsistent rapid ejaculation
**Symptoms**	Ejaculation occurs too early in almost all sexual encounter	New onset of premature ejaculation. Normal ejaculations in the past. Identifiable cause can be found	PE occurs irregularly and is inconsistent	Subjective, self-perception of rapid ejaculation. Ejaculation time is normal
**Quality of ejaculation control**	Ejaculation is rapid throughout lifetime. No ability to control ejaculation	Ability to control ejaculation -diminished or lacking	Ability to control ejaculation - diminished or lacking	Ability to control ejaculation - diminished or lacking
**Treatment **	Pharmacotherapy psychotherapy	Medical management pharmacotherapy psychotherapy education	Reassurance education behavioral therapy	Psychotherapy reassurance education
* Intravaginal ejaculation latency time				

### 5.2. Approach to PE

PE is a clinical diagnosis. Female orgasm can be affected by various factors, and lack of proper history from the female partner may lead to misdiagnosis of female organismic disorder as male partner's PE, again emphasizing the concept of considering sexual partners as a single unit for evaluation and treatment of various sexual problems. The definitions of PE is based on the assumption that female partner is not having any sexual dysfunction like vaginismus or delayed orgasm.

Patients presenting with a history of rapid ejaculation should be asked about the duration of symptoms (primary vs. acquired), about any difficulty in erection, whether he loses erection before ejaculation (PE secondary to ED), whether having rapid ejaculation only during some situation or always/most of the time? (Situational vs. global).

The patient and his partner's perception can be assessed by asking “what is the approximate time between penetration and ejaculation? Is that annoying you or your partner? Is it affecting your relationship? Do you or your partner avoid sexual contact because of this? The answer will give an idea about the severity of the problem and its impact on life.

**Figure 3:**
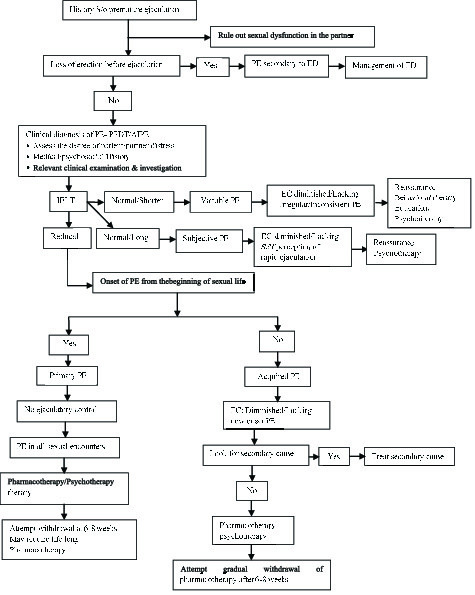
Premature ejaculation management algorithm. S/o: Suggestive of; PE: Premature ejaculation; ED: Erectile dysfunction; PEDT: Premature ejaculation diagnostic tool; AIPE: Arabic index premature ejaculation; IELT: Intravaginal ejaculatory latency time; EC: Ejaculatory control.

In addition, questions like “Do you ejaculate even before penetration? Are you able to delay ejaculation?” will give idea about the perceived degree of control over ejaculation. Also, a stopwatch measurement of IELT will help to quantify the problem (Figure 3).

Detailed history of a previous sexual function and sexual relationship will give a clue about the probable causes in case of secondary or acquired PE.

Moreover, detailed history of other medical problems (like diabetes, hypertension, and hyperthyroidism) and history of intake of medications, trauma or injection will help to identify secondary causes, if there is any.

History of any psychological/psychiatric problems leading to PE and psychological /psychiatric problems secondary to PE must also be assessed and addressed.

Screening questionnaires like premature ejaculation diagnostic tool (PEDT) or arabic index of premature ejaculation (AIPE) along with relevant clinical examination will help diagnose PE in patients with sexual dysfunction (21). Additionally, the index of premature ejaculation (IPE) and male sexual health questionnaire-ejaculatory dysfunction (MSHQ-EjD) can help characterize PE and assess treatment responses.

Several studies have shown that about 80-90% of men with lifelong PE ejaculate within 60 sec and the remaining within 2 min.

The perception about normal ejaculatory latency varies according to country, religious beliefs, and expectation of the patient and his partner, which can also influence the diagnosis of PE.

The clinical examination in patients with PE includes abdominal and genital examination along with neurological examination and examination of lower limb. However, most of the time, these are within normal limits. The clinical examination will also help to reassure the patients that they are anatomically normal. A rectal examination is usually done if PE is associated with painful ejaculation.

## 6. Treatment 

The treatment of PE includes non-pharmacological and pharmacological treatment (Table II).

**Table 2:** Treatment options for premature ejaculation


**Non pharmacological treatment**	**Pharmacological treatment**
Psychosexual counseling	Dapoxetine
Psychotherapy	Topical anesthetic cream
Meditation/relaxation, hypnotherapy	Tramadol
Precoital masturbation	TCA*: Clomipramine
“Stop-start” and	SSRIs**: Paroxetine, Fluoxetine, Serta line
“Squeeze” techniques,	PDE-5 inhibitors***
Use of multiple condoms	**Emerging treatment**
Pelvic floor exercise	Circumcision
Extended foreplay	Dorsal penile nerve cryoablation
Cognitive distraction	Neuromodulation
Alternate sex positions	Hyaluronic acid gel glans augmentation
Interval sex	Botulism toxin injection
Increasing frequency of sex	
Acupuncture	
* Tricyclic antidepressants, ** Selective serotonin reuptake inhibitors, *** Phosphodiesterase (PDE 5) inhibitors	

### 6.1. Non-pharmacological treatment

Psychotherapy and various behavioral therapies are used in the treatment of PE. Psychological therapy requires strong compliance from the partner and is time-consuming, expensive, and less effective than pharmacotherapy and its efficiency decreases with time (22). However, it is the first-line therapy in patient with subjective PE or PE with underlying psychological problem. Psychotherapy is also effective in managing the psychological distress associated with sexual dysfunction. Psychosexual counseling is important to address psychological distress due to PE. Methods like meditation/relaxation, hypnotherapy and neuro-biofeedback help improve ejaculatory control. Various behavioral therapies are also useful in patients with PE which includes masturbation before coitus (precoital masturbation) “stop-start” and “squeeze” techniques, use of multiple condoms, and pelvic floor exercise (23).

The `Stop-start' method, proposed by Semans in 1956 and “squeeze” techniques, by Masters and Johnson in 1970 are the two commonly used techniques for PE.

#### 6.1.1. “Squeeze” technique 

Identifying the “points of no return” (i.e., the point where one feels that ejaculation is imminent and inevitable) during intercourse is an important step in “stop-start” and “squeeze” techniques. In the squeeze technique, the patient holds the penis with thumb and forefinger at the “points of no return” in such a way that the thumb comes over the frenulum and the two forefingers on the opposite side where glans meet the shaft of the penis and squeeze for few seconds. This sustained pressure applied to glans penis results in the contraction of bulbospongiousus muscle via bulbospongiosus reflex leading to diminished ejaculatory urgency, loss of desire to ejaculate and reduced rigidity of penis, thereby helping to prolong sexual activity. Stopping the motion of intercourse and performing sustained contraction of the pelvic floor muscles can also result in diminished ejaculatory urgency which is described as “internal squeeze” without manual pressure (24). In men with pelvic floor muscle dysfunction, the pelvic floor therapy significantly increase IELT, making it a safe and effective treatment option in PE (25).

#### 6.1.2. “Stop-start” technique

In this technique, the partner stimulates the penis until the patient feels the urge to ejaculate. At this point, the patient should stop stimulation and wait for the sensation to pass. Then, they have to stimulate again till the “points of no return” and stop it, thereby helping to prolong sexual activity. Subsequently, the “Stop-start” technique can be practiced during intercourse.

Pelvic floor exercises/Kegel exercises help strengthen pelvic floor muscles. It also helps to control ejaculation. Wearing condom during intercourse reduces the sensitivity and helps delay ejaculation in patients with PE. Other behavioral techniques include extended foreplay, cognitive distraction, alternate sex positions, interval sex, and increasing the frequency of sex. Acupuncture is also proposed to be effective in improving IELT.

### 6.2.Pharmacological treatment

#### 6.2.1. Topical anesthetic cream

Topical anesthetic cream containing lidocaine and pilocarpine applied to penis about 20 min before sexual intercourse increases IELT from 1.49 to 8.45 min (26). Topical eutectic-like mixture for PE, which is a metered dose aerosol containing lidocaine and pilocarpine, also improves IELT 2.4 times than the baseline and is easy to use (27) (Table III).

However, it can cause numbness of genitalia of males, and sometimes because of contamination of cream in vagina, the partner can also have numbness during intercourse (28). Use of condom can prevent transvaginal absorption. Use of topical anesthetic preparations can also be associated with loss of pleasure, orgasm, and erection.

**Table 3:** Drugs used to treat Premature Ejaculation


**Drugs used to treat PE**					
**Drug**	**Dose (mg/day)**	**Half-life (hr)**	**Mean fold increase in IELT**	**Side effects**	**Remarks**
**Topical anesthetic agents**					
**Aerosols and creams containing lignocaine, lignocaine/prilocaine or herbal-derived anesthetic agents**	Apply 20-30 min prior to intercourse	1-2	4-6	ED, loss of sensation in penis and in partner's vagina, skin irritation	Apply to the glans penis well ahead of intercourse. Use of condoms prevents numbness in the partner's genitals
**Opioid**					
**Tramadol**	25-50 mg, 3-5 hr prior to intercourse	5-7	4-7.3	Nausea, dizziness insomnia, dyspepsia, seizures	Significantly increases IELT, heightens sexual satisfaction, and improves ejaculatory control. Risk of opioid addiction, abuse/dependence and serotonin syndrome. Contraindicated with TCAs and SSRIs
**Serotonergic tricyclic antidepressant**					
**Clomipramine **	25-50 25 mg, 4-24 hr prior to intercourse	19-37	4.6-6	Nausea, dry mouth, ED, hot flushes, arrhythmias	2-3 weeks needed for therapeutic effect
**SSRI antidepressants**					
**Paroxetine **	10-40 (20 mg, 3-4 hr prior to intercourse)	21	8.8-11.6	Insomnia, anxiety, nausea, loss of libido, ED, anhidrosis fatigue, nausea, diarrhea, dry mouth and decreased libido Serotonin syndrome	2-3 weeks needed for therapeutic effect
**Sertraline **	25-200 50 mg, 4-8 hr prior to intercourse	26	4.1-5	
**Fluoxetine **	5-20	36	3.9-5	
**Dapoxetine**	30-60 mg 1-3 hr prior to intercourse	1.5	2.5-3.0	Headache, somnolence, dizziness	Effective treatment for both acquired and lifelong PE. Use with caution in patients with cardiac, hepatic, or renal impairment
**Other drugs**					
**Phosphodiesterase-5 inhibitors**	3-6	Headache, flushing, dyspepsia	Does not affect IELT but may improve PE in patients with co-morbid erectile dysfunction by providing a perception of greater control over ejaculation
**Alpha-1 adrenoceptor antagonist (alpha-1 blocker)**					
**Tamsulosin**	0.4 mg	6-9 hr	Postural hypotension, dizziness	
**Silodosin**	4-8 mg	13.3 ± 8.07	3	Retrograde ejaculation, dizziness, diarrhea, orthostatic hypotension, headache, nasopharyngitis, nasal congestion	
PE: Premature ejaculation, ED-Erectile dysfunction, IELT: Intra-vaginal ejaculation latency time, TCAs: Tricyclic antidepressants, SSRIs: Selective serotonin reuptake inhibitors					

#### 6.2.2. Tramadol

Tramadol is commonly used as an analgesic. It acts as a μ-opioid agonist and an inhibitor of reuptake of 5 hydroxytryptamine (5 HT) and nor-adrenaline (NA). It is useful in the management of PE, through a multimodal mechanism that involves inhibition of reuptake of 5 HT and NA, blockade of nociceptive effects, and inhibition of spinal somatosensory-evoked potentials. Because of the short half-life (1.7 hr) and rapid absorption, it can be used as “on demand” tablet (56 mg 2 hr prior to the intercourse) for the treatment of PE (29). It significantly increases IELT, heightens sexual satisfaction, and improves control over ejaculation.

#### 6.2.3. Serotonergic antidepressants: Tricyclic antidepressants (TCA) and selective serotonin reuptake inhibitors (SSRIs)

##### 6.2.3.1. Tricyclic antidepressants (TCA)

TCA inhibit norepinephrine transporter (NET) and serotonin transporter (SERT) and reduce the uptake of noradrenaline and 5-HT by adrenergic and 5-HT neurons. Serotonin inhibit ejaculation, while TCA and SSRIs potentiate its effect, and hence are useful in the management of PE. Among TCAs, clomipramine is used as an “off label drug” to treat PE. Other TCAs are not commonly used to treat PE because of its potential side effect profile (30).

##### 6.2.3.2. Selective serotonin reuptake inhibitors (SSRIs)

SSRIs like paroxetine, fluoxetine, sertraline increases IELT by reducing the uptake of serotonin via blockade of 5-HT transporters and used as an “off label drug” to treat PE (31).

#### 6.2.4. Dapoxetine

Dapoxetine is a potent SSRI and the first molecule specifically developed for the treatment of PE. It binds to 5HT, nor-epinephrine (NE) and dopamine (DA) reuptake transporters and inhibits their uptake in the order of potency NE<5HT>>DA, thereby increasing 5HT level in the synaptic cleft (Figure 2).

The chemical structure is Dapoxetine ((+)-(s)-N, N-dimethyl-A- {2-(1-naphthalenyloxy) ethyl} -benzene methenamine) hydrochloride. Dapoxetine is absorbed rapidly (1-3 hr), the maximum plasma concentration is reached after 1-2 hr with a half-life of 60-80 min, and is eliminated almost completely within 24 hr, and therefore can be used as “on demand drug” for the treatment of PE. Dapoxetine should be swallowed with one glass of water to avoid bitter taste. It is metabolized by multiple enzymes including cytochrome P450 isoforms and flavin monoxygenase-1 and excreted in urine, after metabolism (32). When co-administered with potent CYP2D6 or CYP3A4 inhibitors, the level of Dapoxetine increases.

The recommended initial dose of Dapoxetine is 30 mg, taken as needed 1-3 hr before sexual activity once in every 24 hr and may be increased to a maximum dose of 60 mg daily based o tolerability if 30 mg is insufficient to produce desired therapeutic effects. However, if Dapoxetine is taken `on demand'1-3 hr before sexual activity, it should not be taken again before 24 hr of intake. With 30 mg Dapoxetine the mean IELT increase from 0.9-1.1 min to 2.8-3.9 min and with 60 mg to 3.3-4.2 min compared to placebo (1.8-2.4 min) (33-35). The common side effects of Dapoxetine include nausea, diarrhea, headache, dizziness, insomnia, somnolence, fatigue, and nasopharyngitis. It rarely causes erectile dysfunction. Dapoxetine is found to be safe in therapeutic dosage. It does not cause prolongation of QT interval or increase the risk of significant arrhythmia. Dapoxetine should not be taken with alcohol Since Dapoxetine can produce orthostatic hypotension and syncope, patients are advised to avoid rapid position change within 3 hr of intake of the tablet. An orthostatic test can be done before starting Dapoxetine to assess the risk.

Also, Dapoxetine withdrawal does not produce significant symptoms in contrast to other SSRI withdrawal that produce symptoms such as dizziness, headache, nausea, vomiting, diarrhea, and occasionally agitation, impaired concentration, depersonalization, and irritability.

It is advised to attempt gradual withdrawal of drugs in selected patients, especially in those with acquired PE after six-eight weeks of treatment.

#### 6.2.5. Phosphodiesterase-5 (PDE5) inhibitors 

PDE-5 inhibitors do not increase IELT; however, in patients with PE, it increases the perception of ejaculatory control, ejaculatory confidence, and overall sexual satisfaction (36).

In patients with concomitant PE and ED, PE can be secondary to ED. In such cases, ED should be treated first and PE usually improves with improvement in ED.

PDE-5 inhibitors reduce the refractory time to achieve second erection after ejaculation. Some studies have shown that a combination of PDE-5 inhibitor along with SSRIs increases IELT and sexual satisfaction compared with SSRIs alone, however, it is associated with a higher side effects profile, like headaches and ﬂushing (37). The proposed mechanisms for the benefits of PDE-5i in the treatment of PE includes (a) a central effect resulting in increased NO and reduced sympathetic tone, (b) relaxation of the smooth muscles of vas deferens and seminal vesicles; and (c) reduction in anxiety associated with sexual performance. However, till now, there is no clear evidence on a combination of PDE-5 inhibitors and SSRIs in patients with PE without ED.

#### 6.2.6. Alpha-1 adrenoceptor antagonist (alpha-1 blocker)

Alpha-1 blockers are useful for the treatment of patients with concomitant PE and lower urinary tract symptoms (LUTS). Alpha blocker, tamsulosin, which is commonly used to treat benign prostatic hyperplasia showed a significant inhibit on the emission phase of ejaculation due to decreased contractility of the seminal vesicle or the vas deferens. This increased the ejaculatory threshold, resulting in the beneficial effect in PE. Tamsulosin also reduces ejaculatory volume. Silodosin, a new alpha-1 blocker with powerful affinity toward the alpha-1A adrenoceptor prolongs IELT with three-fold longer than the baseline (38).

### 6.3. Emerging treatment

**Figure 4:**
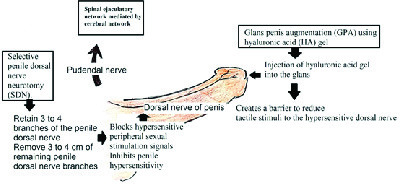
Surgical procedures in the treatment of PE and its mechanism.

Surgical treatment option like circumcision is under investigation. Dorsal penile nerve cryoablation, neuromodulation, and hyaluronic acid gel glans augmentation and botulinum toxin injection into ejaculatory muscles are currently under clinical trial (39, 40) (Figure 4). Other potential future therapeutic options for PE that are under research include Dopamine Receptor Antagonists (selective D3 blockers), GABA-B partial agonists, Serotonin 1A Receptor antagonists, oxytocin receptor antagonists (Epelsiban), Neurokinin-1 receptor antagonists, and Purinergic 2 receptor antagonists.

## 7. Prognosis 

Various data show that the success rate with behavioral and pharmacologic therapy is very high (> 85%) in people with PE. However, the relapse rate is also high and ranges from 20 to 50%. In patients with lifelong PE, IELT will return to pretreatment level within one-three weeks after stopping the medications, whereas in patients with acquired PE, treatment of precipitating factors like ED, sexual performance anxiety will result in sustained improvement in IELT even after stopping medicines like SSRIs.

## 8. Conclusion

PE is a common sexual problem, which cause distress to the patient and his partner. The pathophysiological mechanism is complex. Diagnosis is mainly clinical. Behavioral techniques and psychotherapy have been the main modes of therapy for years. Topical anesthetic creams, TCA and SSRIs tramadol, Alpha-1 adrenoceptor antagonist and PDE-5 inhibitors were the main treatment modalities for years. Development of newer drugs like Dapoxetine have promising role in the management of PE.

##  Conflict of Interest

The authors declare that they have no conflict of interest.
